# D-glucose uptake inhibits bovine alphaherpesvirus 1 post-binding cell process entry via inhibition of PLC-γ1 signaling in a glucose transporter 1-independent manner

**DOI:** 10.1128/spectrum.02456-25

**Published:** 2025-12-11

**Authors:** Xuan Li, Xiuyan Ding, Naifan Zhang, Xiaozhen Ma, Filomena Fiorito, Liqian Zhu

**Affiliations:** 1 College of Life Sciences, Hebei University 162640 , Baoding, China; 2 Department of Veterinary Medicine and Animal Production, University of Naples Federico II 66182, Naples, Italy; Oklahoma State University College of Veterinary Medicine, Stillwater, Oklahoma, USA

**Keywords:** Bovine alphaherpesvirus 1, GLUT1, PLC-γ1, TG, MDBK, Neuro-2A, A549

## Abstract

Bovine alphaherpesvirus 1 (BoAHV-1) is one of the most important viral pathogens responsible for severe economic losses in the cattle industry worldwide. We have previously shown that both phospholipase C gamma1 (PLC-γ1) and β-catenin signaling pathways play crucial roles in BoAHV-1 productive infection. In this study, we demonstrated that BoAHV-1 productive infection in bovine kidney (MDBK) cells and in bovine trigeminal ganglia neurons led to alteration of the protein expression and/or its subcellular localization glucose transporter 1 (GLUT1). In turn, GLUT1 signaling appeared to promote virus productive infection, partially by activating β-catenin-dependent transcriptional activity, as determined using GLUT1-specific small interfering RNAs or the GLUT1-specific inhibitor, BAY-876. Interestingly, D-glucose inhibited the virus post-binding cell entry process, partially through blocking PLC-γ1 signaling. Although GLUT1 is a key glucose transporter, the inhibitory effects of D-glucose on viral entry were independent of GLUT1. Moreover, we showed that D-glucose and the PLC-γ1-specific inhibitor U73122 synergistically inhibited virus cell entry. Collectively, for the first time, we revealed that D-glucose and its potential transporter GLUT1 had opposite effects on BoAHV-1 productive infection (inhibition vs promotion) via manipulation of different cell signaling pathways (PLC-γ1 vs β-catenin). These findings provide novel insights into the mechanisms underlying BoAHV-1 infection involving GLUT1 and glucose and highlight potential therapeutic targets for the development of antiviral strategies.

**IMPORTANCE:**

Virus entry is a complex process that involves the binding of viral glycoproteins to host cell receptors, and various host factors can influence this process. Here, for the first time, we found that D-glucose has the potential to block bovine alphaherpesvirus 1 (BoAHV-1) post-binding cell entry process, possibly through the inactivation of PLC-γ1 signaling. Interestingly, D-glucose regulated PLC-γ1 signaling with a GLUT1-independent mechanism, though D-glucose uptake was partially mediated by GLUT1. We also identified that β-catenin acts as a potential downstream target of GLUT1, which may represent a mechanism regarding how GLUT1 signaling contributes to BoAHV-1 productive infection. Moreover, a distinct GLUT1 staining in the pre-nuclear regions, likely corresponding to the nuclear membrane, was exclusively observed in trigeminal ganglia neurons of latently infected calves, indicating that it is also potentially involved in the virus latency, which deserves further clarification in future studies.

## INTRODUCTION

*Bovine alphaherpesvirus 1* (BoAHV-1), an enveloped DNA virus, is classified within the genus *Varicellovirus* of the subfamily *Alphaherpesvirinae*, in the family *Herpesviridae* (1–3). Following acute infection of mucosal epithelium in the oral, nasal, and ocular cavities, the virus is transported to sensory neurons in the trigeminal ganglia (TG), which serves as a primary site for the establishment of lifelong latency. Reactivation can be frequently triggered by various stressors, leading to viral shedding and subsequent disease development (4, 5), making it difficult to control in herds. Typically, BoAHV-1 infection can induce bovine infectious rhinotracheitis (IBR), conjunctivitis, vulvovaginitis, meningoencephalitis, and abortion, thereby imposing a significant economic burden on the cattle industry (1, 6, 7). For example, the annual cost of BoAHV-1 infection to the US cattle industry is estimated to be around 3 billion dollars (8).

Phospholipase C gamma1 (PLC-γ1) is an enzyme activated by both receptor and non-receptor tyrosine kinases. It hydrolyzes phosphatidylinositol 4,5-bisphosphate to produce critical second messengers, including diacylglycerol (DAG) and inositol-1,4,5-trisphosphate (IP3) (9). DAG mediates the activation of protein kinase C, thereby stimulating a range of downstream signaling pathways, while IP3 mobilizes the release of intracellular calcium (10). In addition to its vital physiological functions, accumulating studies suggest that mutations or aberrant activation of PLC-γ1 play a role in the pathogenesis of various inflammatory diseases (11). For example, a gain-of-function variant in PLC-γ1 has been linked to autoimmunity and systemic inflammation in a patient (12). It has been reported that the activation of PLC-γ1 promotes virus entry (13), but the underlying mechanisms remain to be elucidated.

Glucose transporters (GLUTs) are known to facilitate the cellular uptake of glucose. In mammals, 14 GLUTs have been identified, each with distinct substrate specificities and tissue expression patterns (14). Among them, GLUT1 is the most widely expressed and is a key rate-limiting factor for glucose uptake (15). Beyond its physiological functions, GLUT1 is also associated with infections caused by various herpesviruses, such as Epstein-Barr virus (EBV) (16), Kaposi’s sarcoma-associated herpesvirus (KSHV) (17, 18), and human cytomegalovirus (HCMV) (19). However, the involvement of GLUT1 in BoAHV-1 infection remains unclear. Importantly, it has been reported that dehydroepiandrosterone increases glucose uptake, an effect that can be inhibited by the PLC-γ1 inhibitor U73122 (20), providing a clue that there is a potential interplay between glucose uptake and PLC-γ1 signaling pathway, which warrants further investigation. Therefore, we were interested in exploring whether GLUT1 signaling and glucose uptake had an impact on BoAHV-1 infection and the PLC-γ1 signaling pathway.

In this study, we investigated the involvement of GLUT1 and D-glucose in BoAHV-1 productive infection, as well as the potential regulatory mechanisms, using small interfering RNA (siRNA)-mediated knockdown of GLUT1 protein expression and supplement of D-glucose in cell cultures. Additionally, we explored the effects of viral infection in calf TG neurons on GLUT1 expression using immunohistochemistry (IHC) assays. These findings will enhance our understanding of the mechanisms underlying viral infection, particularly those involving GLUT1 and glucose.

## RESULTS

### Detection of GLUT1 expression in bovine TG neurons during virus acute infection and latency

**Fig 1:**
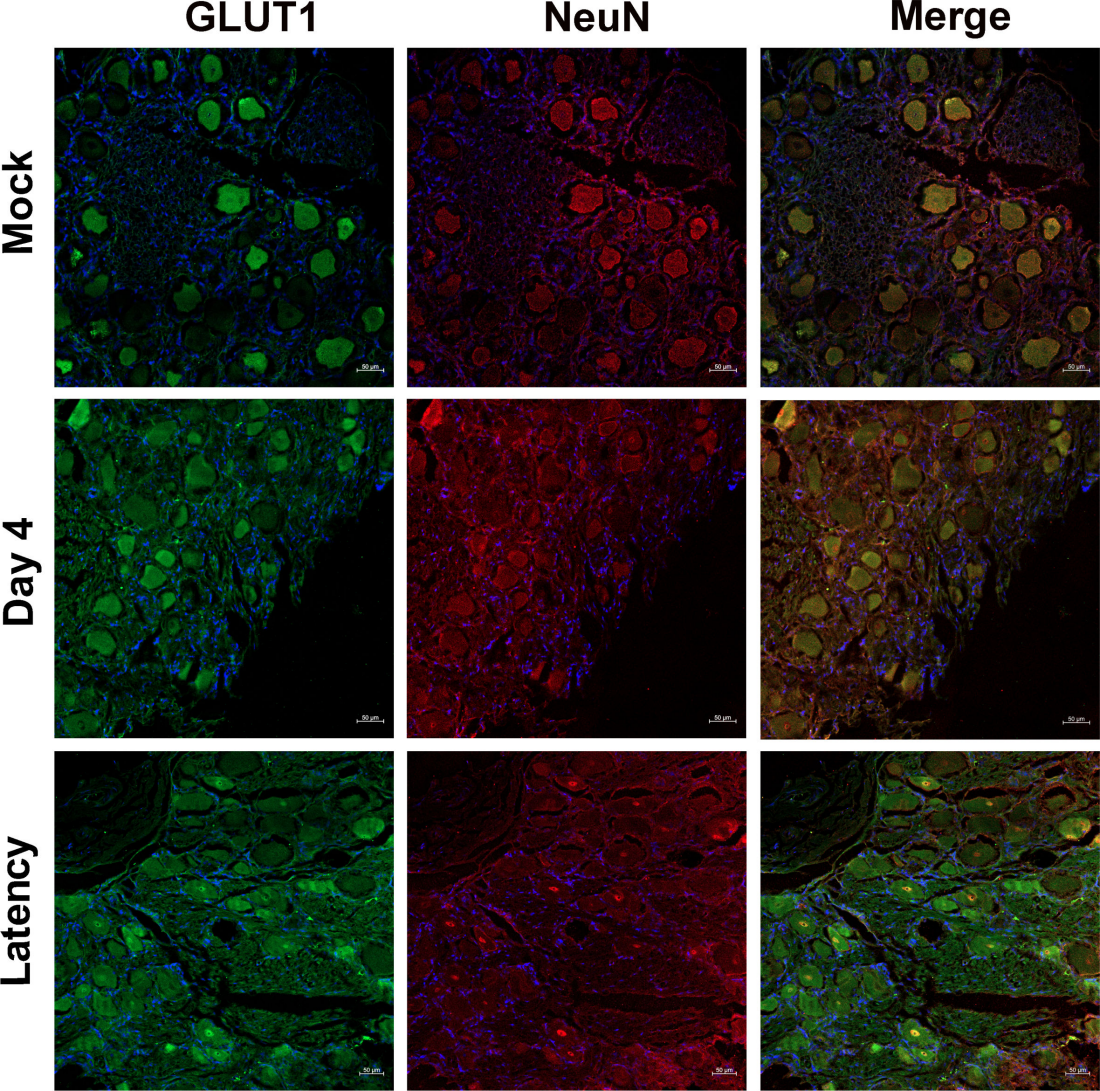
Analysis of the effect of BoAHV-1 infection on GLUT1 expression in bovine TG neurons using IHC. Paraffin-embedded TG sections were incubated with primary antibodies against rabbit anti-GLUT1 and mouse anti-NeuN, followed by incubation with Alexa Fluor 488-conjugated goat anti-rabbit and Alexa Fluor 633-conjugated goat anti-mouse secondary antibodies. Nuclei were labeled with DAPI. Fluorescent signals were visualized, and images were captured using a confocal microscope at 10× magnification. Scale bar: 50 μm.

IHC studies were conducted to gain insights into whether GLUT1 signaling was potentially involved in BoAHV-1 infection in the TG neurons of calves that were either acutely infected (at 4 days post-infection [dpi]) or latently infected (at 60 dpi). As a result, GLUT1 protein expression was readily detected in a subset of TG neurons from both mock-infected and virus-infected calves, including those with acute infection and latency (Fig. 1). However, the GLUT1 expression levels in a population of TG neurons from mock-infected calves were markedly higher than those from the infected calves including that of both acute infection and latency. Interestingly, distinct GLUT1 staining in the perinuclear regions, likely corresponding to the nuclear membrane, was exclusively observed in latently infected calves (Fig. 1, bottom panels).

The neuronal nuclear protein (NeuN) is a neuronal marker. NeuN is broadly expressed in the vertebrate nervous system, which has been used widely as a reliable tool to detect most postmitotic neuronal cell types in neuroscience. To determine whether the stained cells shown are TG neurons, NeuN was detected together with GLUT1 during the immunofluorescence analysis. We found that the staining of GLUT1 and NeuN co-localized and showed a similar profile (Fig. 1), confirming that the immunostained cells by GLUT1 antibody in IHC analysis were TG neurons. However, NeuN was markedly stained in a subset of nuclei in latently infected TG neurons and co-localized with that of GLUT1 (Fig. 1, bottom panels), which suggests that virus infection may also have effects on NeuN localization.

**Fig 2:**
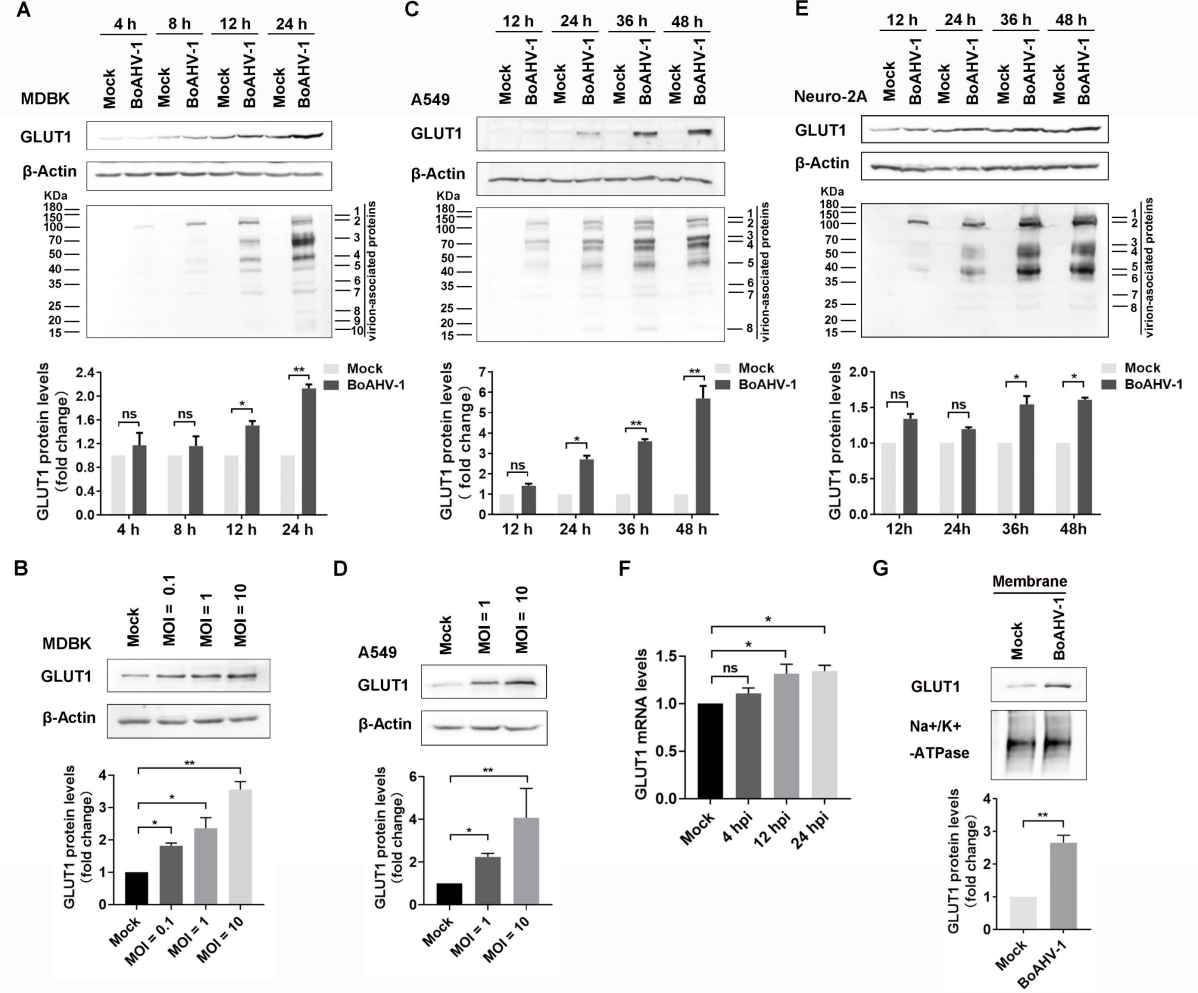
BoAHV-1 productive infection promotes GLUT1 protein expression. (**A, C, and E**) MDBK, A549, and Neuro-2A cells were either mock-infected or infected with BoAHV-1 at an MOI of 1 for indicated time lengths. Then cells were collected and subjected to Western blotting to detect GLUT1 and virion-associated proteins. (**B and D**) MDBK and A549 cells were infected with BoAHV-1 at indicated MOIs ranging from 0.1 to 10, respectively. After 24 h of infection, cell lysates were prepared to detect the GLUT1 protein using Western blotting. (**F**) MDBK cells were infected with virus (MOI of 1) for 24 h, then GLUT1 mRNA levels were examined using RT-qPCR. (**G**) MDBK cells were either mock-infected or infected with virus (MOI of 1) for 24 h, and then membrane fractions were extracted and subjected to Western blotting for the detection of GLUT1. Na^+^/K^+^-ATPase served as a protein loading control for the cytoplasmic membrane fractions. (A, B, C, D, E, and G) The band intensity was analyzed with free software ImageJ. The data shown are means of three independent experiments with error bars indicating SDs. Significance was assessed with a Student’s *t*-test (*, *P* < 0.05; **, *P* < 0.01; ns: not significant).

It is well-known that NeuN expression is indicative of neuron survival, whereas decreased expression of NeuN indicates neuronal death or neuronal incompetence (21), and here, we found that NeuN expression levels were significantly reduced by acute infection in comparison to that in mock-infected TG neurons (Fig. 2, middle panels), indicating that acute viral infection in neurons resulted in marked cell death, which is consistent with a previous report (22).

### BoAHV-1 productive infection increases GLUT1 steady-state protein expression in cell cultures

The potential involvement of GLUT1 in BoAHV-1 productive infection was initially assessed in bovine kidney (MDBK) cells. After infection for 12 and 24 h, GLUT1 steady-state protein expression significantly increased, with protein levels increasing to approximately 1.51- and 2.13-fold, respectively, compared to the mock-infected control (Fig. 2A). When the cells were infected using increasing multiplicity of infection (MOI), ranging from 0.1 to 10 for 24 h, the GLUT1 steady-state protein levels increased in correlation with MOIs, reaching approximately 1.81-, 2.36-, and 3.56-fold, respectively (Fig. 2B). This result confirmed that viral productive infection specifically enhances GLUT1 protein expression in MDBK cell cultures at later stages of viral infection. However, GLUT1 protein levels were not dramatically affected following virus infection at 1 h (data not shown).

BoAHV-1 virus has the capacity to infect a range of cancer cells, including the human lung cancer cell line (A549) and mouse neuroblastoma (Neuro-2A) cells, albeit with lower efficiency than bovine (MDBK) cells (23–27). Here, these cell lines were employed to further investigate the effects of viral infection on GLUT1 protein expression. Infection of A549 cells with BoAHV-1 at an MOI of 1 resulted in increased steady-state expression of GLUT1 protein at 24, 36, and 48 h post-infection (hpi), with protein levels increasing to approximately 2.71-, 3.60-, and 5.70-fold, respectively, compared to the mock-infected control (Fig. 2C). When A549 cells were infected with the virus at increasing MOIs ranging from 1 to 10 for 24 h, GLUT1 protein levels increased in correlation with the MOI, reaching approximately 2.23- and 4.07-fold compared to the mock-infected control at MOIs of 1 and 10, respectively (Fig. 2D). Similarly, in Neuro-2A cells, the steady-state protein expression of GLUT1 also increased following viral infection. Compared to the mock-infected control, the protein levels increased to approximately 1.54-fold at 36 hpi and 1.61-fold at 48 hpi (Fig. 2E). Thus, BoAHV-1 productive infection upregulates GLUT1 protein expression in both A549 and Neuro-2A cells.

Real-time quantitative PCR (RT-qPCR) analysis revealed that in viral-infected MDBK cells, GLUT1 mRNA levels increased approximately 1.31-fold at 12 hpi and 1.34-fold at 24 hpi, compared to mock-infected controls (Fig. 2F). The elevated steady-state protein levels of GLUT1 corroborate these increased mRNA levels in response to viral infection. These findings suggest that viral productive infection may lead to increased GLUT1 protein expression, at least in part, via upregulation of mRNA expression.

As a GLUT, GLUT1 is abundantly expressed in the cell membrane, where it performs essential functions (28). When the cell membrane fractions were isolated via using specific commercial kits and subjected them to Western blotting analysis, we found that viral infection significantly increased the accumulation of GLUT1 protein in the plasma membrane fractions. Specifically, the levels of GLUT1 in virus-infected fractions were elevated to approximately 2.65-fold compared to the mock-infected fractions (Fig. 2G). These results suggest that viral infection enhances the accumulation of GLUT1 in the cell membrane, potentially affecting its biological function.

**Fig 3:**
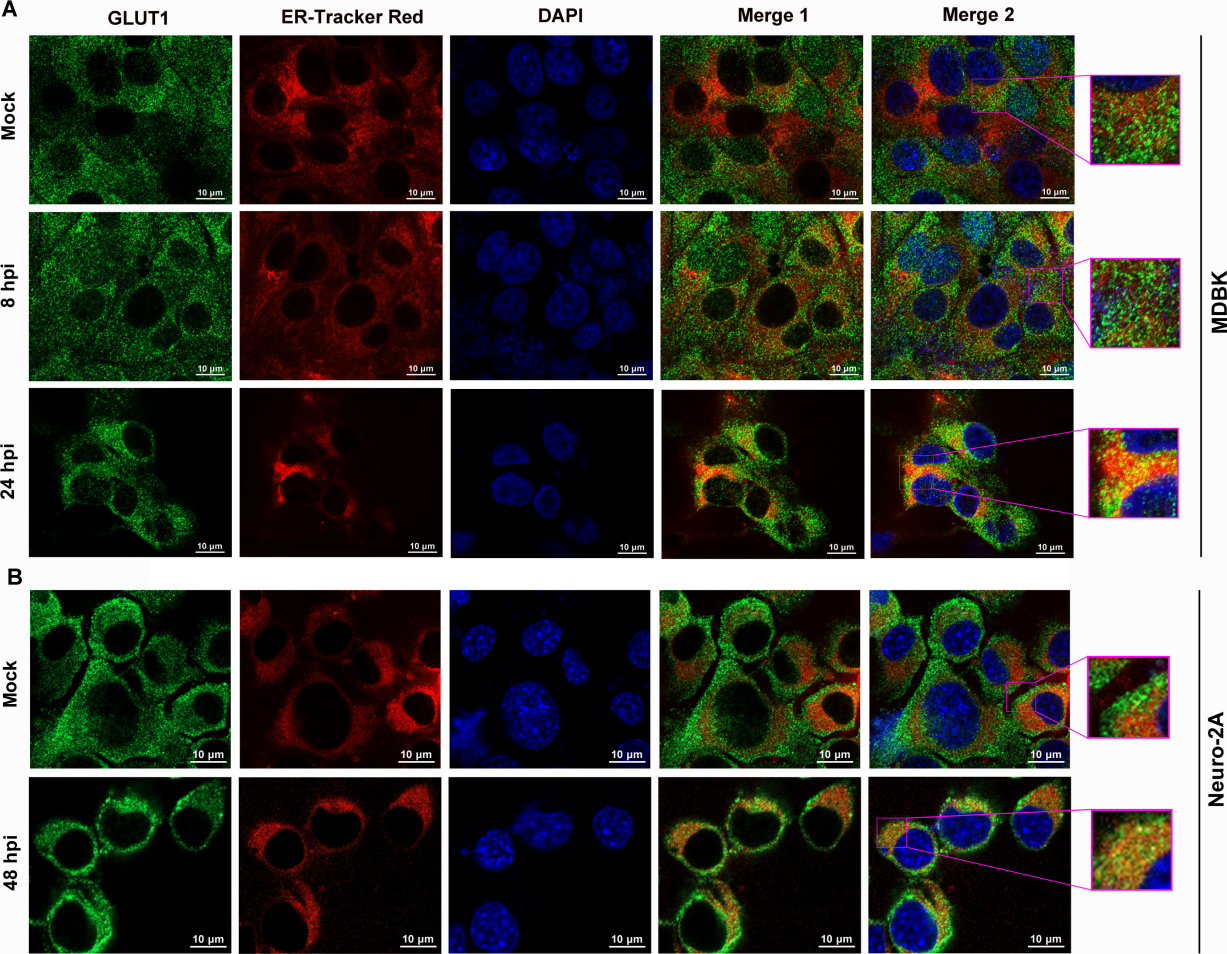
Immunofluorescence detection of GLUT1 protein co-localization with the ER in cells infected with BoAHV-1. (**A**) MDBK cells were either mock-infected or infected with BoAHV-1 (MOI=1) for 8 and 24 h. (**B**) Neuro-2A cells were mock-infected or infected with BoAHV-1 (MOI=1) for 48 h. Then the ER of the cells was fluorescently labeled (red), and GLUT1 protein was immunostained via IFA assay using GLUT1-specific antibody (green). The nuclei were stained using DAPI (blue). Images were captured using a confocal microscope. The data shown are representations of three independent experiments. Scale bar: 10 μm.

The endoplasmic reticulum (ER) is the site where ribosomes synthesize proteins that are destined for secretion, insertion into the plasma membrane, or incorporation into other organelles (29). We observed that the accumulation of GLUT1 protein in the ER was significantly increased in MDBK cells following viral infection for 24 h, compared to both the mock-infected control and the levels at 8 hpi (Fig. 3A, zoom-in areas). Similarly, in Neuro-2A cells, the ER accumulation of GLUT1 was significantly higher after 48 h of viral infection than that in the mock-infected control (Fig. 3B, zoom-in areas). The enhanced accumulation of GLUT1 in the ER correlates with the increased steady-state protein levels during productive viral infection at later stages. Thus, BoAHV-1 productive infection upregulates GLUT1 protein expression across various cell types.

### BoAHV-1 virion-associated proteins co-localize with GLUT1 during virus productive infection at later stages

**Fig 4:**
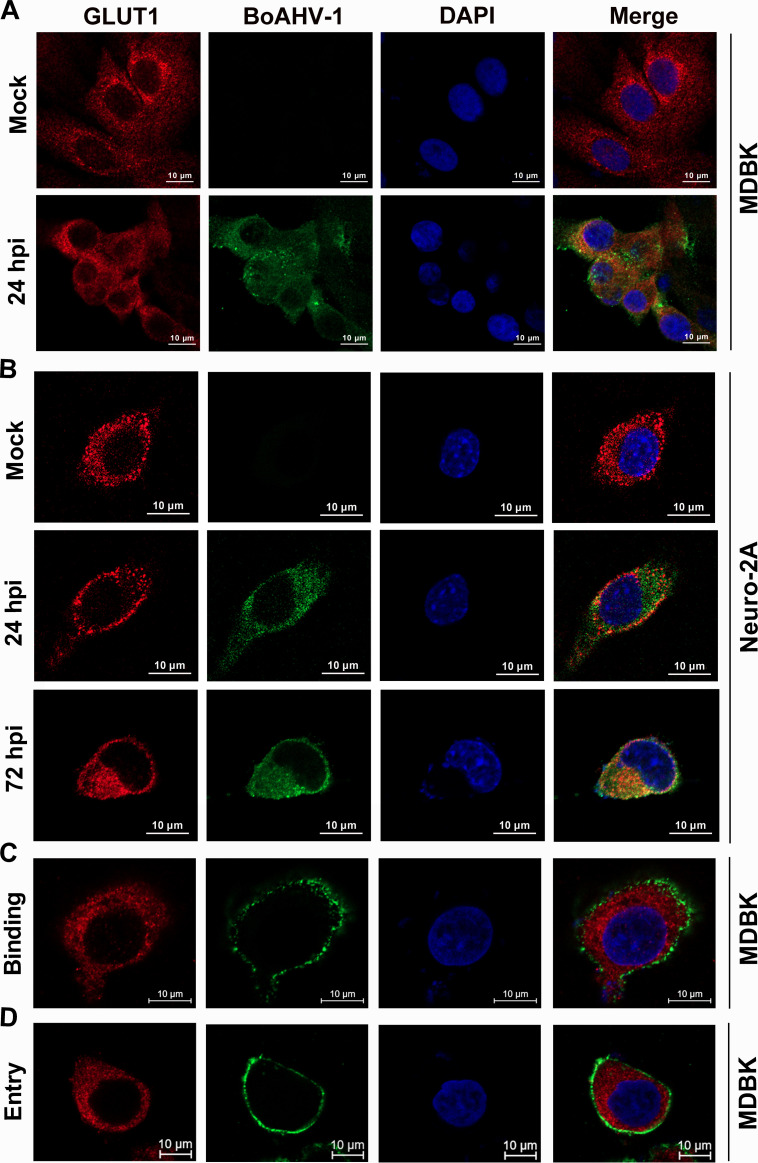
Detection of GLUT1 and virion-associated proteins in BoAHV-1 infected MDBK and Neuro-2A cells using IFA. MDBK (A, C, and D) and Neuro-2A cells were either mock-infected or infected with BoAHV-1 at an MOI of 1 for different durations under designated conditions. Specifically, MDBK cells were infected for 24 h at 37°C to allow for virus productive infection (**A**), for 1 h at 4°C to allow for virus binding to the cells (**C**), or for 1 h at 37°C to allow for virus binding and subsequent entry (**D**). Neuro-2A cells were infected for 24 and 72 h at 37°C (**B**). Then these cells were subjected to immunostaining using GLUT1-specific antibody (red) and anti-BoAHV-1 serum (green). The cell nuclei were stained with DAPI (blue). Images were then captured using a confocal microscope. Scale bars: 20 μm.

**Fig 5:**
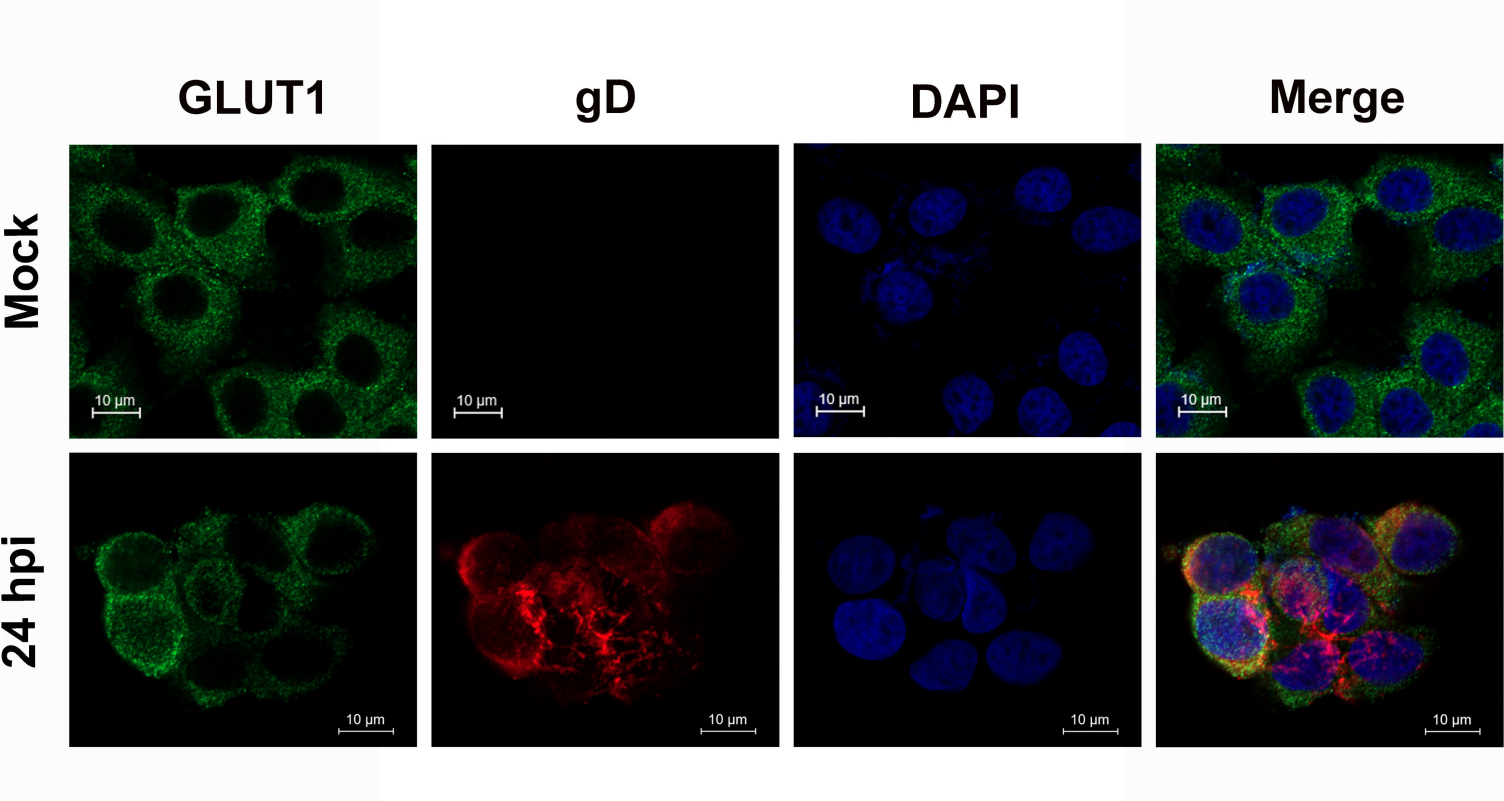
Detection of GLUT1 and viral protein gD in BoAHV-1 infected MDBK cells using IFA. MDBK cells were either mock-infected or infected with BoAHV-1 at an MOI of 1 for 24 h at 37°C. Subsequently, the cells were subjected to immunostaining using antibodies against GLUT1 and BoAHV-1 viral protein gD, respectively. The nuclei were stained with DAPI. Images were captured using a confocal microscope. Scale bars: 20 μm.

To investigate whether viral infection influenced the subcellular distribution of GLUT1, both MDBK and Neuro-2A cells were infected, followed by immunofluorescence assay (IFA) using an antibody specific to GLUT1 and a commercially available serum produced by immunization of purified BoAHV-1 virions. As a result, GLUT1 was readily detected in both cell lines, regardless of viral infection (Fig. 4A and B). However, dramatic alterations in GLUT1 localization were not observed in MDBK cells following viral infection for 24 h (Fig. 4A) or in Neuro-2A cells following viral infection for either 24 or 72 h (Fig. 4B). Nonetheless, a subset of GLUT1 co-localized with virion-associated proteins in MDBK cells at 24 hpi and in Neuro-2A cells at both 24 and 72 hpi (Fig. 4A and B). Interestingly, when MDBK cells were incubated with the virus in ice-cold medium to allow virus binding but not cell entry (Fig. 4C), or when the cells were subsequently transferred to 37°C to allow virus entry to proceed (Fig. 4D), no co-localization between virions and GLUT1 was observed. These findings suggested that the *de novo* virion-associated proteins might associate with GLUT1 protein during the later stages of virus infection, providing a hint that GLUT1 may be involved in the virus replication cycles at the later stage of viral infection. Furthermore, when we performed IFA assays using antibodies against GLUT1 and the viral protein gD, we observed that GLUT1 co-localized with viral protein gD (Fig. 5), which further confirmed that GLUT1 co-localizes with virion-associated proteins.

### GLUT1 plays an important role in BoAHV-1 productive infection

**Fig 6:**
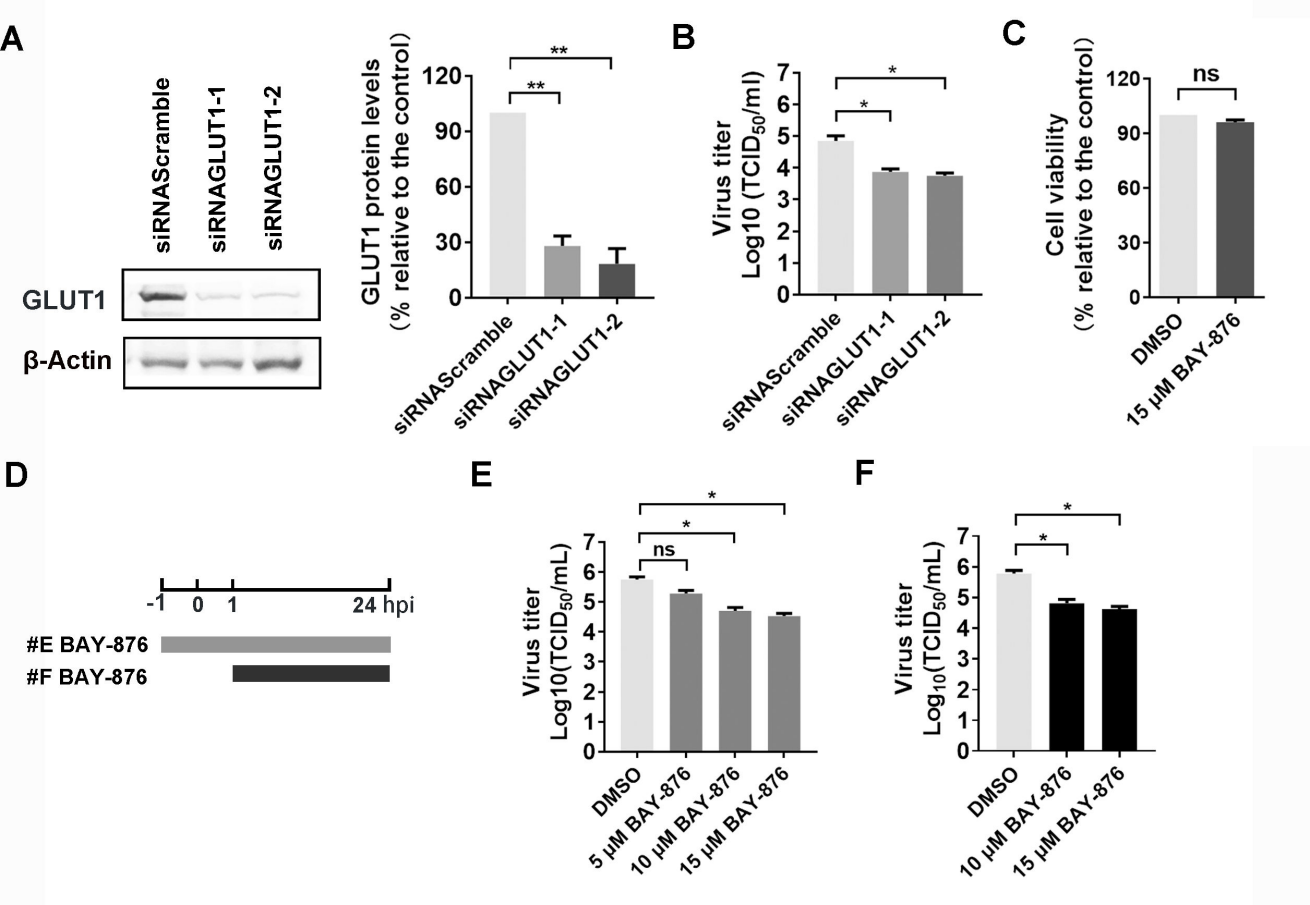
GLUT1 plays an important role in BoAHV-1 productive infection. (**A**) MDBK cells in six-well plates were transfected with either scrambled siRNA (150 pmol) or two individual siRNA targeting GLUT1 (150 pmol), referred to as siRNAGLUT1-1 and siRNAGLUT1-2, respectively. At 48 h post-transfection, GLUT1 protein levels were detected via Western blot. The band intensity was analyzed with free software ImageJ. (**B**) MDBK cells in six-well plates were transfected with 150 pmol of scrambled siRNA, siRNAGLUT1-1, and siRNAGLUT1-2, respectively. After transfection for 36 h, the cells were infected with BoAHV-1 (MOI = 1) for 1 h. After three washes with PBS, fresh medium was replaced. At 24 hpi, the virus yield in the cell cultures was measured, with results expressed as TCID_50_/mL. (**C**) The cytotoxicity of BAY-876 (15 μM) in MDBK cells for 24 h was analyzed by Trypan-blue exclusion test. (**D**) Diagram showing two different experimental conditions. (**E**) BAY-876 treatment from −1 to 24 hpi. (**F**) BAY-876 treatment from 1 to 24 hpi. (**E**) MDBK cells in 24-well plates pretreated with either DMSO control or BAY-876 at indicated concentrations for 1 h were infected with BoAHV-1 (MOI = 1) for 1 h in the presence of the indicated chemicals. After three washes with PBS, fresh media containing either DMSO control or BAY-876 were added for further incubation. At 24 hpi, the virus titers were measured, with results expressed as TCID_50_/mL. (**F**) MDBK cells in 24-well plates were infected with BoAHV-1 (MOI = 1) for 1 h. After three washes, fresh media containing respective chemicals were added for further incubation. At 24 hpi, the virus titer was determined with results expressed as TCID_50_/mL. The results shown are means of three independent experiments, with error bars indicating SDs. Significance was assessed with Student’s *t*-test (*, *P *< 0.05; **, *P* < 0.01; ns, not significant).

To investigate the roles of GLUT1 played in BoAHV-1 productive infection, GLUT1-specific siRNAs were employed for this study. Two commercially available siRNAs, referred to as siRNAGLUT1-1 and siRNAGLUT1-2, could significantly knock down GLUT1 protein expression (Fig. 6A, left panels). Compared to the scrambled siRNA control, GLUT1 protein expression reduced to approximately 27.95% and 18.22% by siRNAGLUT1-1 and siRNAGLUT1-2, respectively (Fig. 6A, right panels). When GLUT1 protein expression was knocked down by these siRNAs and the cells were subsequently infected with the virus for 24 h, the viral yields were significantly reduced by approximately 0.97- and 1.09-log, respectively, relative to the scrambled siRNA control (Fig. 6B). Thus, these data indicated that GLUT1 signaling may play an important role in BoAHV-1 productive infection in MDBK cells.

Reports indicate that BAY-876 is a highly potent and selective inhibitor of GLUT1 compared to other GLUTs (30). To test whether it has effects on viral productive infection, MDBK cells were treated with either vehicle control DMSO or 15 μM of BAY-876, a concentration that does not cause cytotoxicity as determined by the Trypan blue exclusion assay (Fig. 6C), as described elsewhere (31). The treatment of cells was carried out at different times and durations of virus infection, as shown in the diagram (Fig. 6D). The pharmacological inhibition of GLUT1 with BAY-876 resulted in a reduction of viral growth in MDBK cells. Specifically, when the virus-infected cells were treated with the inhibitor during the infection plus a 1-h pretreatment, viral titers reduced by approximately 1.05- and 1.22-log with 10 and 15 μM of BAY-876, respectively (Fig. 6E). When virus-infected cells were treated with the inhibitor from one hpi and maintained until the end of the virus infection (at 24 hpi), viral titers were reduced by approximately 0.95- and 1.15-log with 10 and 15 μM of BAY-876, respectively (Fig. 6F). Thus, these findings confirm that GLUT1 plays a critical role in BoAHV-1 productive infection in MDBK cells, at least by affecting viral replication at post-entry stages.

### GLUT1 stimulates β-catenin-dependent transcription, which may contribute to viral productive infection

**Fig 7:**
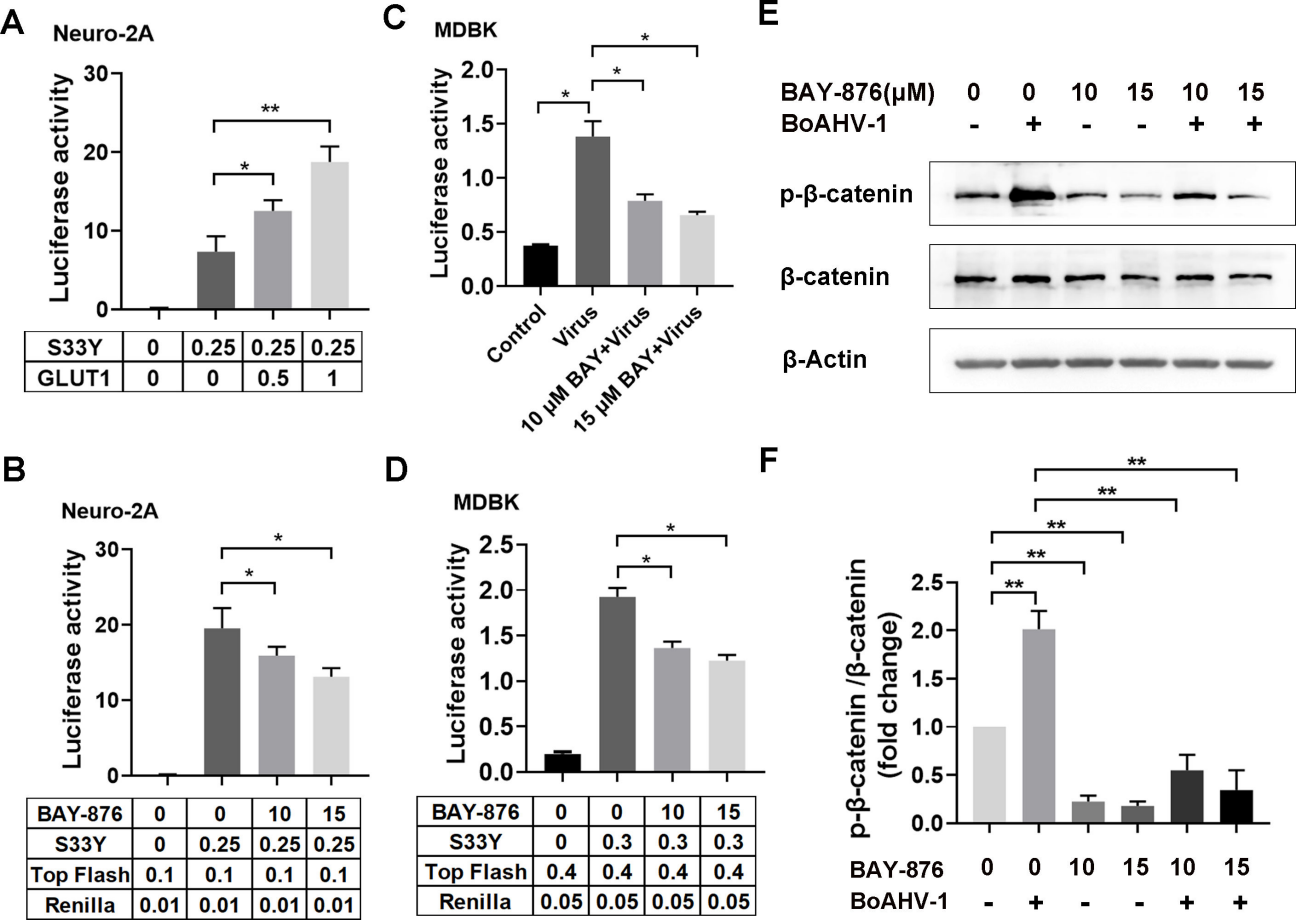
GLUT1 stimulates β-catenin-dependent transcription. (**A**) Neuro-2A cells were co-transfected with indicated plasmid at the designed dose using Lipofectamine 3000. At 48 h after transfection, cell lysates were prepared and subjected to dual luciferase assays. (**B and D**) Neuro-2A and MDBK cells were co-transfected with indicated plasmid at the designed dose using Lipofectamine 3000. At 24 h post-transfection, the cells were treated with BAY876 at indicated concentrations for 24 h. Then cell lysates were prepared and subjected to dual luciferase assays. (**C**) MDBK cells in 12-well plates were co-transfected with indicated plasmid at the designed dose using Lipofectamine 3000. After transfection for 24 h, the cells were infected with BoAHV-1 (MOI = 1) for 24 h along with treatment of either DMSO control or BAY-876 at the designated concentrations. Then cell lysates were prepared and subjected to dual luciferase assay. (**A–D**) The data shown are raw values of firefly luciferase activity normalized to that of *Renilla* luciferase (**E**) MDBK cells in 60 mm dishes were pretreated with BAY876 at indicated concentrations for 1 h, after which they were mock-infected or infected with BoAHV-1 at an MOI of 1 in the presence of BAY876 or DMSO control. After the infection for 24 h, the cell lysates were prepared for Western blotting to detect the protein levels of p-β-catenin (S552) and β-catenin. (**F**) The band intensity was analyzed with free software ImageJ. The data shown are means of three independent experiments with error bars indicating SDs. Significance was assessed with a Student’s *t*-test (*, *P* < 0.05; **, *P* < 0.01; ns: not significant).

BoAHV-1 productive infection stimulates β-catenin-dependent transcription and the ability of β-catenin to stimulate cell survival and cell cycle regulatory factors enhances viral productive infection in non-neuronal cells (32, 33). Interestingly, it has been proven that shRNA-mediated suppression of GLUT1 protein expression leads to an increase in β-catenin protein levels (15), implying that GLUT1 negatively regulates β-catenin protein expression. However, the effect of GLUT1 on β-catenin-dependent transcriptional activity, indicative of β-catenin activity, has yet to be reported. Consequently, we investigated the influence of GLUT1 on β-catenin-dependent transcription using a luciferase assay by transfection of Neuro-2A cells, as described elsewhere (32). In rationale, the activated β-catenin in the nucleus can interact with a T-cell factor (TCF) family member bound to a consensus site (5′-T/A-T/A-CAAAG-3′) (34). β-catenin binding to TCF displaces bound co-repressors and recruits a panel of transcriptional coactivators, thus stimulating promoters containing TCF binding sites. The plasmid Super 8× TOPFlash contains eight TCF binding sites upstream of a minimal promoter that drives firefly luciferase reporter expression and thus accurately measures β-catenin dependent transcription (33). Neuro-2A cells were selected for this study because they are highly amenable to transfection, and β-catenin is rarely detectable in these cells using Western blot, making it an ideal cell line for β-catenin plasmid transfection assay (33). Our findings revealed that β-catenin-dependent transcription was enhanced when co-transfected with the GLUT1 plasmid in a dose-dependent manner (Fig. 7A). Inconsistently, the β-catenin-dependent transcriptional activity was significantly decreased by GLUT1-specific inhibitor BAY-876 in a dose-dependent manner (Fig. 7B). To test whether GLUT1 is potentially involved in the activity of β-catenin-dependent transcription stimulated by virus infection in MDBK cells, the plasmid Super 8× TOPFlash was transfected and then infected with virus in the presence of GLUT1-specific inhibitor BAY-876 at various concentrations. As above, the β-catenin-dependent transcriptional activity stimulated by virus productive infection was significantly diminished by BAY-876 at concentrations of both 10 and 15 μM (Fig. 7C). In the context of mock infection, β-catenin-dependent transcriptional activity in MDBK cells was also inhibited by BAY-876 at concentrations of both 10 and 15 μM (Fig. 7D). Thus, GLUT1 may positively regulate β-catenin-dependent transcriptional activity stimulated by either activated β-cateninS33Y or virus productive infection.

It has been reported that phosphorylation of β-catenin at Ser552 (S552) increases its transcriptional activity (35). Thus, the effects of BAY-876 on the protein levels of p-β-catenin (S552) were detected in the presence or absence of viral infection. We found that the protein levels of p-β-catenin (S552) in MDBK cells decreased following treatment with BAY-876, regardless of virus infection (Fig. 7E and F). These data corroborate our findings that GLUT1 is potentially involved in the activation of β-catenin-dependent transcriptional activity, a potential mechanism to stimulate the virus productive infection.

### Glucose significantly inhibits virus post-binding cell entry process

**Fig 8:**
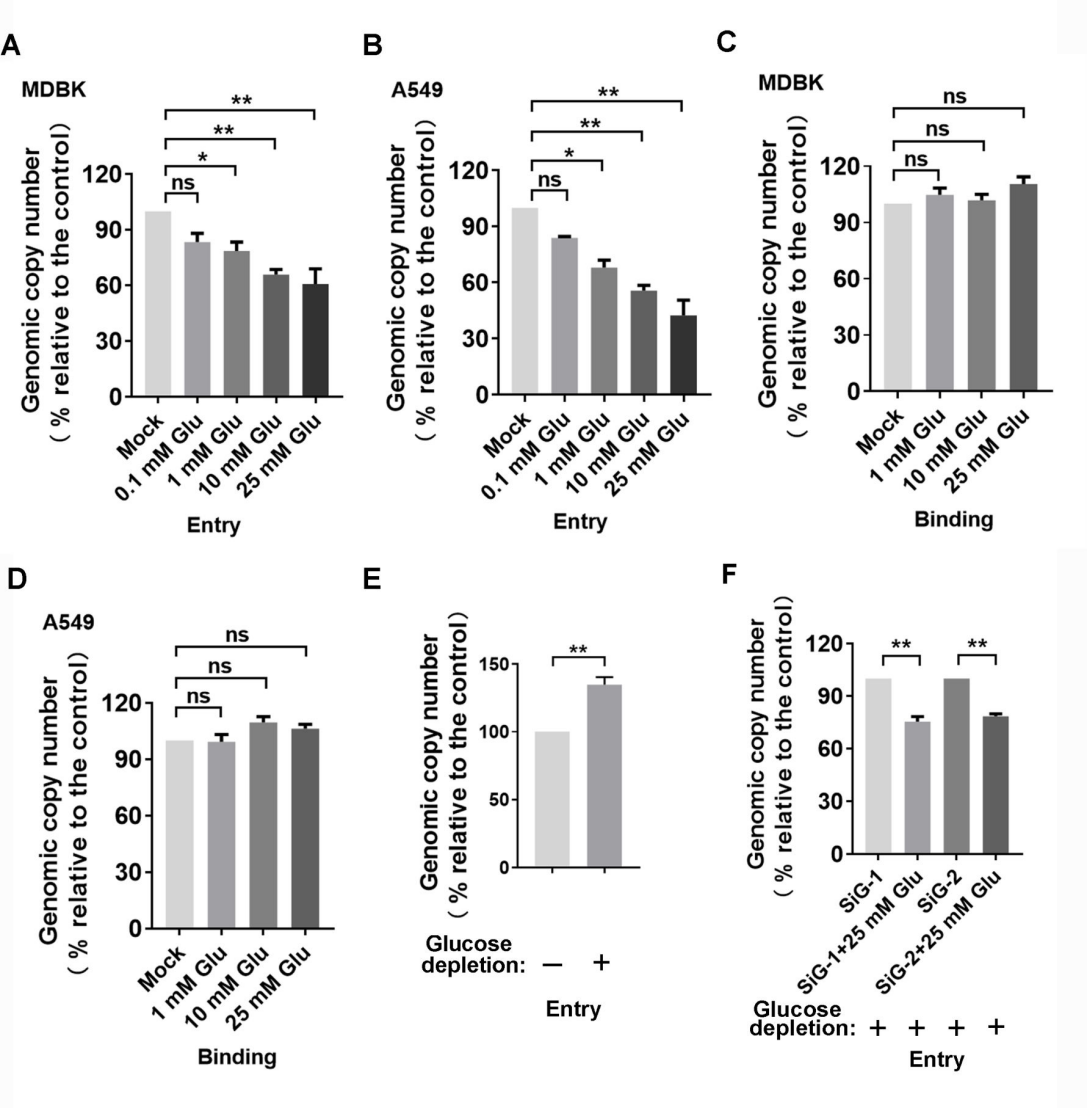
Determine the effects of D-glucose uptake on BoAHV-1 entry. (**A and B**) MDBK (A) and A549 (B) cells were subjected to glucose depletion using glucose-free DMEM for 12 h. The cells were then infected with BoAHV-1 (MOI = 1) in the presence of D-glucose at increasing concentrations ranging from 0.1 to 25 mM at 37°C for 30 min. After three washes, genomic DNA was then purified and subjected to quantitative assessment of the viral genome. (**C and D**) MDBK (**C**) and A549 (**D**) cells were subjected to glucose depletion using glucose-free DMEM for 12 h. The cells were washed twice with ice-cold PBS and then infected with BoAHV-1 (MOI = 1) in the presence of D-glucose at increasing concentrations ranging from 0.1 to 25 mM at 4°C for 30min to allow virus binding to the cells without entry. After three washes, genomic DNA was purified and subjected to quantitative assessment of cell-bound viral genome. (**E**) MDBK cells were subjected to glucose depletion using glucose-free DMEM for 12 h. Then infected with BoAHV-1 (MOI = 1) at 37°C for 30 min. After washing three times with PBS, genomic DNA was purified using commercial DNA purification kits, and the cell-associated viral genome was quantitatively evaluated. (**F**) MDBK cells in six-well plates were transfected with 150 pmol of siRNAGLUT1-1 and siRNAGLUT1-2, respectively. At 36 h post-transfection, the cells were subjected to glucose depletion using glucose-free DMEM for 12 h, followed by BoAHV-1 infection (MOI = 1) in the presence of 25 mM D-glucose and kept at 37°C for 30 min. After three washings, genomic DNA was purified, and the cell-associated viral genome was quantitatively evaluated using qPCR. The results shown are the mean of three independent experiments, with error bars indicating SDs. Significance was assessed with Student’s *t*-test (*, *P* < 0.05; **, *P* < 0.01; ns, not significant).

The binding and transport of glucose across the plasma membrane is a rapid process (36), which can be dependent on or independent of GLUTs such as GLUT1, depending on the specific tissue, cellular context, and metabolic conditions (37). We were interested in whether glucose uptake affects viral infection. To minimize the effects of glucose in the cell culture medium, MDBK cells were subjected to glucose depletion by culturing them in D-glucose-free Dulbecco’s modified Eagle medium (DMEM) for 12 h. They were then infected with BoAHV-1 in the presence of D-glucose at specified concentrations for 30 min at 37°C, allowing virus entry and glucose uptake to occur simultaneously. Subsequently, the cell-associated viral genomes were quantified using qPCR. We observed that D-glucose significantly reduced the amount of cell-associated viral genome in a dose-dependent manner in both MDBK and A549 cells (Fig. 8A and B). Specifically, in MDBK cells, the amount of cell-associated viral genome decreased to approximately 78.64%, 65.94%, and 60.74% with D-glucose supplementation at concentrations of 1, 10, and 25 mM, respectively, compared to the control in the absence of D-glucose (Fig. 8A). A549 cells exhibited similar trends as observed in MDBK cells, with viral genome copy numbers reduced to 68.12%, 55.64%, and 42.49% with D-glucose at concentrations of 1, 10, and 25 mM, respectively (Fig. 8B). These findings suggest that the D-glucose uptake inhibits viral entry process. However, when both MDBK and A549 cells were incubated with the virus at 4°C, the amount of cell-associated virions, indicative of virus-binding, was not significantly affected by D-glucose supplementation (Fig. 8C and D). Collectively, these results indicated that D-glucose uptake inhibits the virus post-binding cell entry process. This was further confirmed by the observation that D-glucose depletion leads to increased levels of virus entry, as assessed in MDBK cells (Fig. 8E).

Given that GLUT1 is potentially involved in D-glucose uptake, we investigated whether GLUT1 is associated with D-glucose uptake-mediated inhibition of viral entry. We found that supplementation with D-glucose led to decreased levels of viral entry in GLUT1-depleted MDBK cells via siRNA transfection. Specifically, viral genomic levels were reduced to approximately 75.32 and 78.48% in cells transfected with siRNA-GLUT1 and siRNA-GLUT2, respectively, by 25 mM D-glucose relative to the vehicle control (Fig. 8F). Therefore, it is highly likely that the inhibition of viral entry mediated by D-glucose uptake is not mechanically dependent on GLUT1.

### Glucose uptake significantly inhibits PLC-γ1 signaling

**Fig 9:**
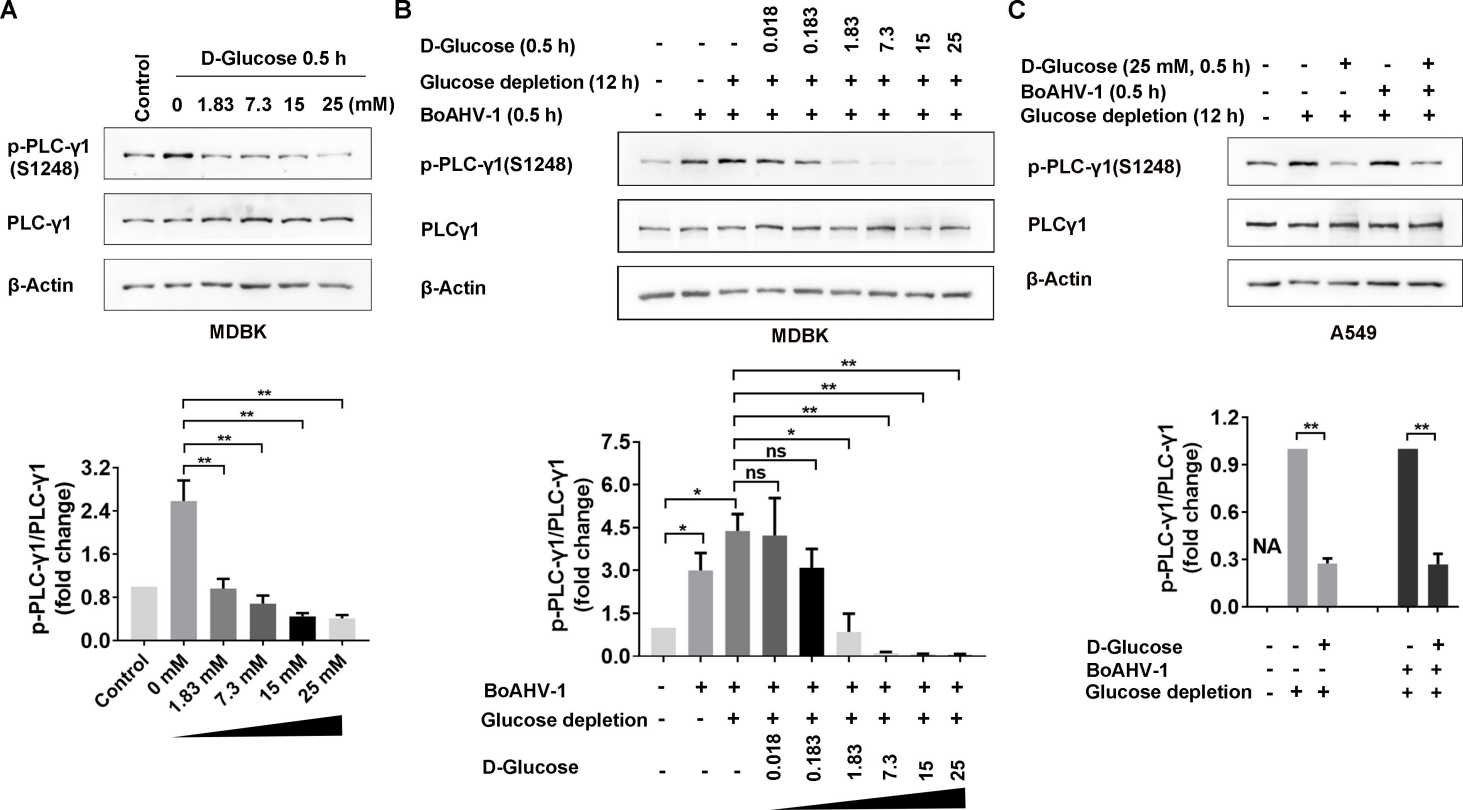
Glucose affects phosphorylation of PLC-γ1 at Ser1248. (**A**) MDBK cells were subjected to glucose depletion using D-glucose-free DMEM or mock depletion using high glucose DME for 12 h. The cells were then treated with D-glucose at the indicated concentrations in glucose-free DMEM for 0.5 h, after which cell lysates were prepared. The protein levels of p-PLC-γ1(Ser1248) and PLC-γ1 were detected by Western blotting. (**B**) MDBK cells were subjected to glucose depletion or mock depletion as described above. The cells were then infected with BoAHV-1 (MOI =1) for 0.5 h in the presence of D-glucose at the indicated concentrations in glucose-free DMEM. After the infection, the lysates were prepared. The protein levels of p-PLC-γ1(Ser1248) and PLC-γ1 were detected by Western blotting. β-Actin was used as a loading control and subsequent quantitative analysis. (**C**) A549 cells in 60 mm dishes were subjected to glucose depletion via culturing cells using glucose-free DMEM. Then, the cells were infected with BoAHV-1(MOI =1) for 0.5 h in the presence of D-glucose at a concentration of 25 mM in glucose-free DMEM. After the infection, the cell lysates were prepared. The protein levels of p-PLC-γ1(Ser1248) and PLC-γ1 were detected by Western blotting. The band intensity was analyzed with free software ImageJ. The results shown are the average of three independent experiments, with error bars indicating SDs. Significance was assessed with Student’s *t*-test (*, *P* < 0.05; **, *P* < 0.01; ns, not significant).

We have previously established that the activation of the PLC-γ1 signaling pathway is pivotal for BoAHV-1 entry, with the mechanism remaining to be elucidated (13). We sought to investigate whether glucose uptake affects the activation of PLC-γ1 signaling via detection of the protein levels of phosphorylated PLC-γ1 at Ser1248 [(p-PLC-γ1(S1248)]. To this end, MDBK cells were subjected to glucose depletion for 12 h using D-glucose-free DMEM. The cells were then treated with increasing concentrations of D-glucose, reintroduced into the glucose-free DMEM, for 30 min to evaluate the subsequent effects on PLC-γ1 signaling. Notably, D-glucose depletion resulted in a significant increase in p-PLC-γ1 (S1248) protein levels compared to those in cells that did not undergo D-glucose depletion. Quantitative analysis indicated that p-PLC-γ1 (S1248) protein levels increased to approximately 2.58-fold following D-glucose depletion (Fig. 9A). The reintroduction of D-glucose resulted in a dose-dependent dephosphorylation of p-PLC-γ1 (S1248). Quantitative analysis revealed that p-PLC-γ1 (S1248) protein levels decreased to 37.21%, 26.36%, 17.44%, and 15.89% following treatment with D-glucose at concentrations of 1.83, 7.3, 15, and 25 mM, respectively, compared to the control supplemented with phosphate-buffered saline (PBS) (Fig. 9A). Thus, D-glucose uptake leads to the inhibition of PLC-γ1 signaling in MDBK cells.

We have previously reported that BoAHV-1 infection leads to an increase of PLC-γ1(S1248) levels as early as 30 min post-infection (13). Here, further investigations were performed to determine whether this activation is influenced by D-glucose uptake. Our findings indicated that BoAHV-1 infection for 30 min significantly elevated p-PLC-γ1(S1248) protein levels in MDBK cells, regardless of glucose depletion (Fig. 9A, upper panels). Specifically, p-PLC-γ1 (S1248) protein levels increased approximately 3.0-fold and 4.39-fold in response to viral infection in a condition either with or without D-glucose, respectively, compared to mock-infected control (Fig. 9B, lower panels). However, virus infection-induced PLC-γ1 phosphorylation at Ser1248 was significantly attenuated by treatment with D-glucose at the concentrations of 15 and 25 mM. Consequently, only minimal detectable levels of p-PLC-γ1 (S1248) protein were observed (Fig. 9B).

Similar to the observations in MDBK cells, D-glucose depletion significantly increased p-PLC-γ1 (S1248) protein levels in A549 cells, regardless of whether the cells were infected with the virus (for 30 min). Conversely, the addition of D-glucose inhibited p-PLC-γ1(S1248) protein levels in these cells, irrespective of viral infection (Fig. 9C, upper panels). Quantitative analysis revealed that the addition of 25 mM D-glucose in A549 cells reduced p-PLC-γ1(S1248) protein levels to approximately 27.42% and 26.83%, relative to that of the vehicle control, regardless of virus infection (Fig. 9C, lower panels). Therefore, D-glucose exhibits a strong ability to inhibit the PLC-γ1 signaling pathway in A549 cells, regardless of viral infection. Collectively, these results suggest that D-glucose uptake may suppress PLC-γ1 signaling pathway, a potential mechanism to inhibit virus entry processes.

### Inhibition of virus post-binding cell entry by PLC-γ1(S1248) inhibitor U73122 is further enhanced by D-glucose

**Fig 10:**
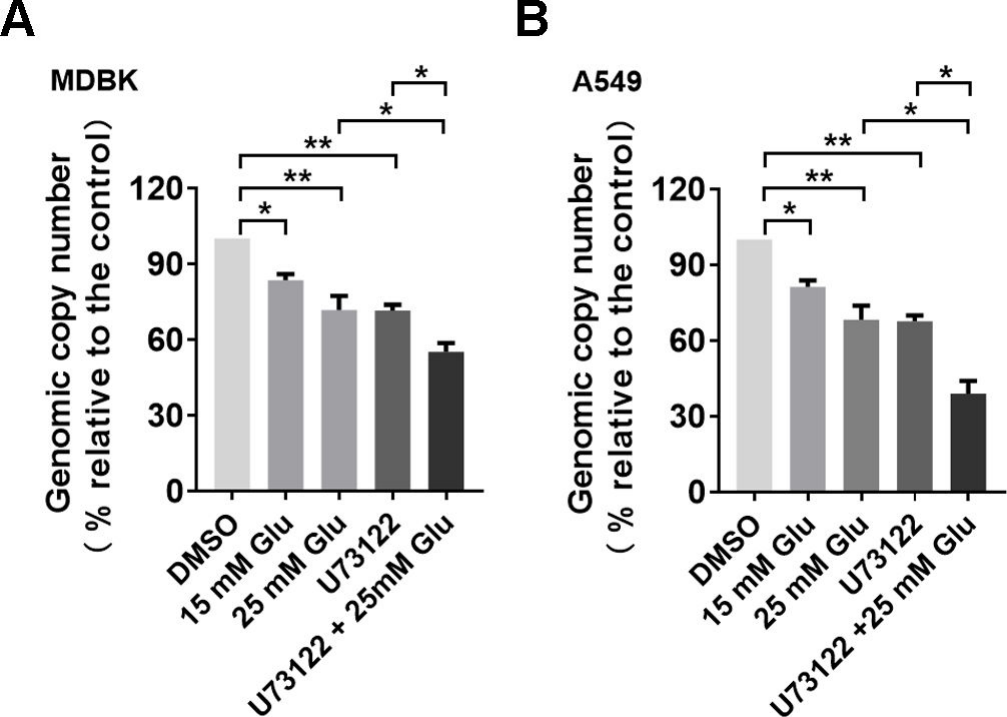
The effects of U73122 and glucose on BoAHV-1 entry process. (**A and B**) MDBK (**A**) and A549 (**B**) cells in six-well plates were subjected to glucose depletion via culturing the cells using glucose-free DMEM for 12 h. The cells were infected with BoAHV-1 (MOI = 1) in the presence of either U73122 or D-glucose alone, or in combination, at the designated concentrations, at 37°C for 30 min. After extensive washes, genomic DNA was extracted, and the viral genome bound to the cells was quantitatively evaluated using qPCR. The data shown are the means of three independent experiments with error bars representing the SDs. The significance was evaluated by Student’s *t*-test (*, *P* < 0.05; **, *P* < 0.01).

PLC-γ1 signaling has been evidenced to play an important role in BoAHV-1 entry process (13), and our findings demonstrated that glucose treatment inhibits PLC-γ1 signaling, which corroborates its inhibitory effects on the virus post-binding cell entry processes (Fig. 8 and 9). We wondered whether D-glucose inhibited viral entry also via a PLC-γ1-independent manner. For this purpose, both MDBK and A549 cells were processed for virus binding at 4°C, and then they were transferred into a condition at 37°C to allow virus entry to occur in the presence of glucose. Then, the cell-bound but not penetrated viral particles were removed by washing with citric acid buffer, as described elsewhere (38). This treatment allowed only penetrated viral genome to be detected by qPCR. As expected, the amount of penetrated virions in MDBK cells was significantly reduced by U73122, with levels decreasing to 71.55% relative to the dimethyl sulfoxide (DMSO) control. Furthermore, the amount of viral genome entering the cells was further reduced by U73122 when combined with D-glucose, with viral genome levels decreasing to 55.13% relative to the DMSO control (Fig. 10A). Similar inhibitory effects of U73122 on virus entry were also observed in A549 cells. Specifically, the levels of viral genomes entering the cells were reduced to approximately 67.63% by U73122 alone and to 39.05% by the combination of U73122 and D-glucose, relative to the DMSO control (Fig. 10B). These data suggest that the inhibitory effects of U73122 on virus post-binding entry were further enhanced by D-glucose.

## DISCUSSION

Glucose metabolism is a sophisticated process through which cells transform glucose into ATP and other metabolic intermediates, which is the initiation of glucose uptake, primarily mediated by GLUTs such as GLUT1, a key rate-limiting step in the glycolysis pathway (15). It is well established that the interplay between viral infection and glucose metabolism has been extensively studied across a spectrum of viruses, including SARS-CoV-2 (39), respiratory syncytial virus (40), coxsackievirus B3 (41), human immunodeficiency virus 1 (HIV-1) (42), HCMV (43), KSHV (44), herpes simplex virus 1 (45), EBV (46, 47), and Zika virus (48). These viruses employ diverse mechanisms to regulate glucose uptake and GLUT1 expression. For instance, HIV-1 infection increases both glucose uptake and GLUT1 expression (42), whereas HCMV infection increases glucose uptake but downregulates GLUT1 expression (43).

In this study, we demonstrated that BoAHV-1 productive infection in numerous cell cultures leads to a concordant increase in GLUT1 protein expression (Fig. 2), which is supported by the increased accumulation of GLUT1 protein in the ER (Fig. 3). Importantly, we showed that GLUT1 plays a critical role in BoAHV-1 productive infection, as evidenced by experiments using both siRNAs and the GLUT1-specific inhibitor, BAY-876 (Fig. 6). We further revealed that GLUT1 protein positively regulates β-catenin-dependent transcription, as evidenced by a luciferase assay (Fig. 7A through D), and GLUT1-specific inhibitor BAY-876 significantly reduced the phosphorylation of β-catenin at Ser552 (Fig. 7E), indicative of transcriptionally activated β-catenin (35). Since β-catenin’s ability to stimulate cell survival and cell cycle regulatory factors plays an important role in viral productive infection (32), GLUT1-mediated activation of β-catenin-dependent transcription may represent a novel mechanism underlying the contribution of GLUT1 to virus replication. Of note, it has been reported that β-catenin plays an important role in stimulating GLUT1 expression, with a range of host factors involved, such as CD36 (49), the Zic family of proteins (50), and the nuclear pyruvate kinase M2, a coactivator of β-catenin (51). Thus, we propose that there is a potential reciprocal regulatory loop between GLUT1 and β-catenin: β-catenin promotes GLUT1 protein expression, and in turn, GLUT1 stimulates β-catenin-dependent transcription, which warrants further investigation to validate this hypothesis in future studies. Interestingly, IFA assay indicated that a portion of virion-associated proteins co-localized with GLUT1 during the later stages of viral productive infection, but not during the binding or post-binding cell entry stages (Fig. 4). This may represent a novel mechanism underlying the role of GLUT1 in regulating productive viral infection, which warrants extensive investigation in the future.

Accumulating studies suggest that glucose enhances viral infection through various mechanisms. For example, it has been demonstrated that high levels of glucose promote SARS-CoV-2 infection via a HIF-1α/glycolysis-dependent pathway (52), or through the involvement of cathepsin L (53). Elevated glucose levels also enhance Senecavirus A replication, in part by dampening interferon-related antiviral signaling (54). Blood glucose levels have been shown to facilitate dengue virus infection in the mosquito *Aedes aegypti* (55), while glucose influences hepatitis C virus release (56). It has been reported that high glucose levels induce the downregulation of silent information regulator 1, leading to the epigenetic transactivation of KSHV lytic genes (57). However, a study indicates that a lower glucose concentration (5 mM) significantly promotes hepatitis C virus replication via enhanced transcription and autophagy compared to higher glucose concentrations (10 and 25 mM) (58). In our study, we discovered that elevated glucose levels, such as 25 mM, significantly inhibit the virus post-binding cell entry process (Fig. 6A through D). This inhibition is likely due to the suppression of PLC-γ1 signaling (Fig. 9), which is known to be crucial for viral entry (13). Consequently, it appears that D-glucose may exert distinct effects on viral infection across various viruses. Of note, although GLUT1 may be involved in D-glucose transport, the inhibition of virus infection mediated by D-glucose uptake is not dependent on GLUT1 (Fig. 8F). Of note, 14 GLUTs have been identified in mammals, each with distinct substrate specificities and tissue expression patterns (14). And it has been reported that 2-NBDG and 6-NBDG, analogs of D-glucose, can bind to and enter mammalian cells via transporter-independent mechanisms (36). So whether other GLUTs or passive diffusion is involved in the transport of glucose, and consequently regulate PLC-γ1 signaling, is an interesting area that deserves extensive studies in the future.

We have previously reported that activated PLC-γ1 signaling plays a crucial role in the trafficking of virions from the Golgi apparatus to the plasma membrane, as well as in subsequent virus release (59). In this study, we discovered that higher concentrations of D-glucose lead to reduced levels of p-PLC-γ1 (S1248) (Fig. 9), which may serve as a potential mechanism to negatively regulate the virus post-binding cell entry process (Fig. 8). Moreover, the inhibitory effects of the PLC-γ1-specific inhibitor U73122 on the virus post-binding cell entry process are further enhanced by D-glucose (Fig. 10). These findings may provide another novel perspective on understanding the role of PLC-γ1 in the virus productive infection. Consequently, PLC-γ1 exerts multiple effects on virus productive infection, making it a promising therapeutic target for the development of novel antiviral drugs. These observations suggest that D-glucose analogs may have the potential to be developed into antiviral drugs for treating BoHAV-1 infection, particularly when used in combination with inhibitors of the PLC-γ1 signaling pathway. This warrants intensive investigation in future studies.

**Fig 11:**
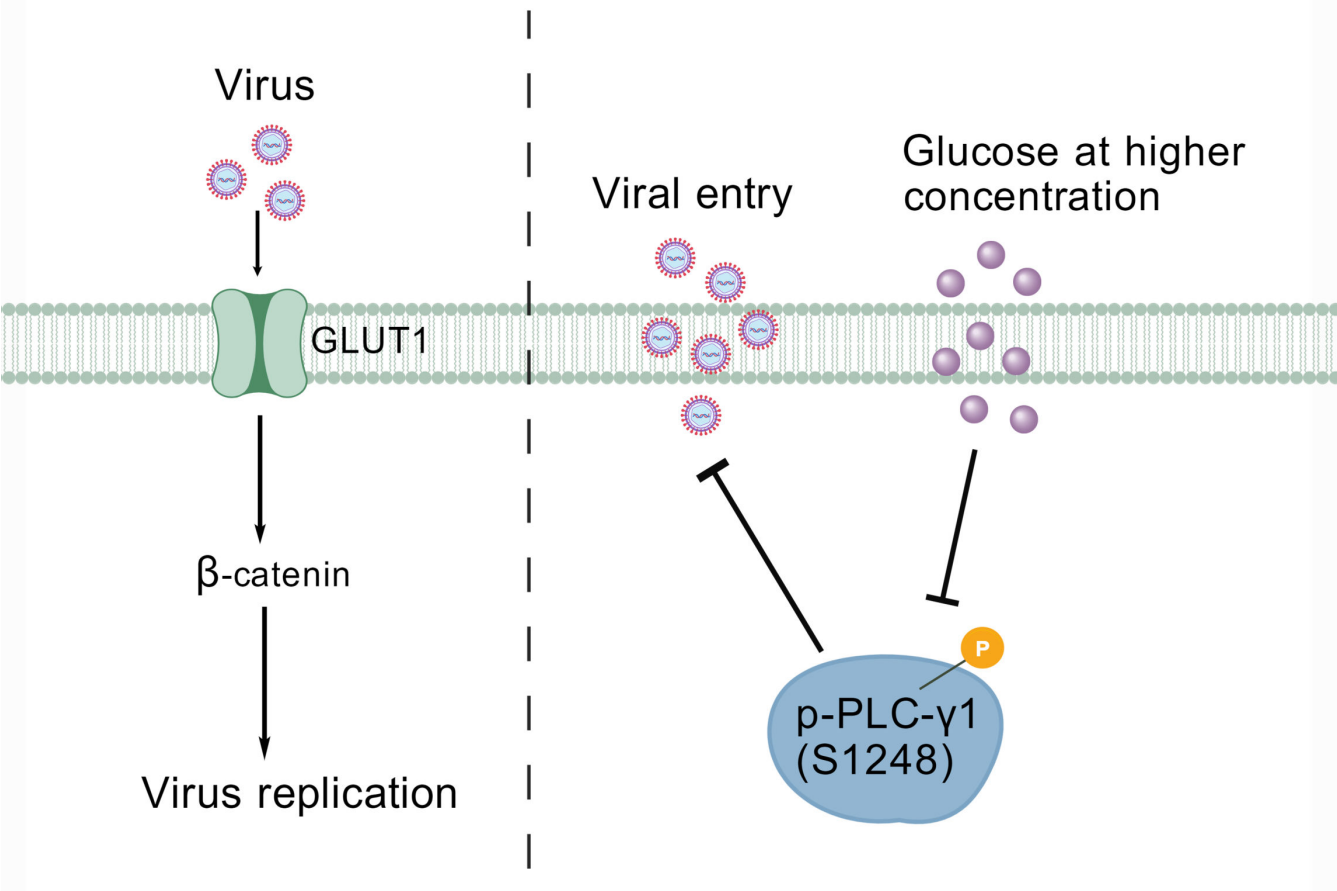
Schematic of putative model on the functional effects of GLUT1 and D-glucose on BoAHV-1 productive infection. GLUT1 plays an important role in the stimulation of β-catenin-dependent transcriptional effects, a potential mechanism to affect viral productive infection. D-glucose at higher concentration is capable of inhibiting virus entry partially via blocking PLC-γ1 signaling as demonstrated by reduced phosphorylation of p-PLC-γ1 at Ser1248. The graphic was created by the online program of BioGDP.com (60).

Based on our observation, we draw a mechanism model as demonstrated in Fig. 11 that GLUT1 may promote viral replication partially via stimulation of β-catenin signaling pathway and D-glucose uptake inhibits PLC-γ1 signaling and consequently leads to blocking virus entry process. Although GLUT1 is a critical transporter of glucose, D-glucose uptake inhibits the virus post-binding cell process entry via a mechanism that is independent of GLUT1. For the first time, we revealed that D-glucose uptake suppresses the PLC-γ1 signaling pathway, which may serve as a potential mechanism to inhibit virus entry. Thereby, the mechanism by which D-glucose blocks PLC-γ1 phosphorylation and the functional impacts of D-glucose or GLUT1 modulation on viral infection outcomes *in vivo* remain unexplored, which are fascinating issues that remain to be elucidated in the future.

## MATERIALS AND METHODS

### Cells, viruses, and plasmids

MDBK and A549 cells (purchased from Chinese model culture preservation center, Shanghai, China) were routinely passaged and maintained in DMEM supplemented with 10% fetal bovine serum. Neuro-2A cells were provided as a gift kindly by Dr. Dongli Pan from Zhejiang University. BoAHV-1 strain NJ-16-1 isolated from bovine semen samples (61) was propagated in MDBK cells. Aliquots of virus stocks were stored at −70°C for this study.

The plasmid S33Y, which expresses a constitutively active Flag-tagged β-catenin protein, was a gift from Bert Vogelstein (Addgene plasmid number 16519) (62). The plasmid M50 Super 8× TOPFlash contains a promoter that is stimulated by β-catenin and was a gift from Randall Moon (Addgene plasmid number 12456) (63). The GLUT1-FLAG plasmid expressing wild-type human GLUT1 protein with a C-terminal 3× FLAG tag in pQCXIP vector was a gift from Jason MacGurn (Addgene plasmid # 200094)(64).

### Antibodies and reagents

The following antibodies were used in this study: GLUT1 rabbit polyclonal antibody (pAb) (cat# A11208), PLC-γ1 rabbit pAb (cat# A7711), β-tubulin rabbit pAb (cat# AC015), and β-actin rabbit monoclonal antibody (mAb) (AC026) were bought from Abclonal Technology (Woburn, MA, USA). Phospho-PLCγ1 (Ser1248) rabbit mAb (cat# 8713S), HRP-conjugated goat anti-mouse IgG (cat# 7076), and HRP-labeled goat anti-rabbit IgG (cat# 7074) were purchased from Cell Signaling Technology (Danvers, MA, USA). LaminA/C mouse mAb (cat# sc-376248) was provided by Santa Cruz Biotechnology (Dallas, TX, USA). Goat anti-BoAHV-1 serum (cat# PAB-IBR) was provided by VMRD Inc. (Pullman, WA, USA). Donkey anti-goat IgG H&L (HRP) (ca# ab97110) was provided by Abcam (Cambridge, UK). Alexa Fluor 488-conjugated goat anti-rabbit IgG (H + L) (cat# A-11008) was provided by Invitrogen Life Technologies (Waltham, MA, USA). Phospholipase C (PLC) inhibitor U73122 (cat# HY-13419) and GLUT1-specific inhibitor BAY-876 (cat# HY-100017) were ordered from MedChemExpress (Monmouth Junction, NJ, USA).

### Western blotting analysis

Cell lysates were prepared using RIPA lysis buffer (1× PBS, 1% NP-40, 0.5% sodium deoxycholate, 0.1% SDS) supplemented with protease inhibitor cocktail. These samples were boiled in Laemmli sample buffer for 5 min, then subjected to separation on SDS-PAGE (8% or 10%), and transferred to polyvinylidene fluoride membranes. Immuno-reactive bands were developed using Clarity Western ECL Substrate (Bio-Rad, cat# 1705061).

For the designated studies, band intensity was quantitatively analyzed using the free software Image J program, which was downloaded from the provided linker https://imagej.net/ij/download.html, on 1 December 2020. Significance was assessed with a Student’s *t*-test by using GraphPad Prism software (v8.0). *P* values of less than 0.05 (*P < 0.05) were considered significant for all the calculations.

### Immunofluorescence assay

MDBK cells in eight-well chamber slides (Nunc Inc., IL, USA) were either mock-infected or infected with BoAHV-1 at an MOI of 1. After a 24-h infection, cells were fixed with 4% paraformaldehyde in PBS for 10 min at room temperature, permeabilized with 0.25% Triton X-100 in PBS for 10 min at room temperature, and blocked with 1% bovine serum albumin (BSA) in phosphate-buffered saline with Tween-20 (PBST) for 1 h followed by incubation with GLUT1 antibody in 1% BSA in PBST overnight at 4°C. After three washes, cells were incubated with Alexa Fluor 488-conjugated goat anti-rabbit IgG (H + L) (Invitrogen, cat# A-11,008, 1:1,500 dilution) for 1 h in the dark at room temperature. After three additional washes, the nuclei were stained using DAPI (4′,6-diamidino-2-phenylindole). Slides were covered with coverslips by using antifade mounting medium (Electron Microscopy Sciences, cat# 50-247-04). Images were captured using a confocal microscope (Zeiss).

### Real-time qPCR assay

To analyze the impact of glucose uptake on the virus’s entry process, monolayer MDBK and A549 cells in six-well plates were incubated with glucose-free DMEM for 12 h, followed by infection with BoAHV-1 (MOI = 1) in the presence of D-glucose at concentrations ranging from 25 to 0.1 mM at 37°C for 30 min. After three washes with PBS buffer, cells were collected for total DNA purification using a commercial viral DNA purification kit (Tiangen, cat# DP-348) according to the manufacturer’s instructions. Freshly prepared DNA served as a template for real-time qPCR assay to measure viral DNA levels with primers targeting the BoAHV-1 polymerase gene, and β-Actin DNA analysis was used as an internal control, as described elsewhere (65).

Real-time PCR was conducted using the LightCycler 96 Fast Real-Time System (Roche, CHE). The levels of the viral genome, represented by the BoAHV-1 polymerase gene, were normalized to that of the β-actin gene. The relative levels of the viral genome were calculated using the 2^−ΔΔCT^ method by comparison to the control.

### RNA isolation and quantification of GLUT1 mRNA by qRT-PCR

Total RNA was extracted from the cells using a TRIzol LS Reagent (Ambion, cat#10296010) following the manufacturer’s instructions. Freshly prepared total RNA (1 μg) was used as a template for synthesizing first-strand complementary DNA (cDNA) with commercial random hexamer primers using Thermoscript RT-PCR System Kit (Invitrogen, cat#11146-024) following the manufacturer’s instructions. The cDNA products were then used as templates for real-time quantitative PCR to measure mRNA levels of GLUT1 and 18S rRNA with the following primers: GLUT1 (forward primer: 5′- GTGCTCCTGGTTCTGTTCTTCA −3′, and reverse primer: 5′- GCCAGAAGCAATCTCATCGAA −3′), and 18S rRNA (forward primer 5′- GTAACCCGTTGAACCCCATT-3′, and reverse primer 5′- CCATCCAATCGGTAGTAGCG-3′). Analysis of 18sRNA mRNA was used as an internal control. Real-time PCR was carried out using the LightCycler 96 Fast Real-Time System (Roche, CHE). The relative mRNA level of the GLUT1 gene was calculated using the method (2^−ΔΔCT^) by comparison to the 18sRNA control.

### siRNA transfection assay

MDBK cells in six-well plates were transfected with scrambled siRNA or two GLUT1-specific siRNAs, each at a dose of 150 pmol, provided by Genepharma (Shanghai, China). At 48 h post-transfection, cell lysates were prepared using the cell lysis buffer described above and subjected to Western blot analysis to detect GLUT1 protein levels.

To analyze the effects that GLUT1 has on viral replication, MDBK cells were transfected with siRNAs for 36 h, followed by infection with BoAHV-1 at an MOI of 1 for 24 h. Progeny viruses in MDBK cells were then detected with results expressed as TCID_50_/mL, calculated using the Reed-Muench formula.

### BAY-876 treatment of MDBK cells during virus infection

MDBK cells of confluent in 24-well plates were infected with BoAHV-1 (MOI = 1) along with the treatment of chemical BAY-876 (MCE, cat# HY-100017) at the indicated concentration for 1 h at 37°C. After three washes with PBS, fresh medium with BAY-876 at indicated concentrations was added to each well. After infection for 24 h, viral yields were titrated in MDBK cells, respectively. Cell cultures treated with DMSO were used as a control. The results were expressed as TCID_50_/mL calculated using the Reed-Muench formula.

### Dual-luciferase reporter assay

Neuro-2A cells grown in monolayer in 60-mm dishes were incubated with fresh DMEM for 2 h prior to transfection to reduce the effects of serum in cell culture on the transfection efficacy. The cells were then co-transfected with the Super 8× TOPFlash plasmid, S33Y, a plasmid encoding Renilla luciferase under the control of a minimal herpesvirus thymidine kinase (TK) promoter (Promega), along with or without a GLUT1 plasmid using Lipofectamine 3000 Transfection Reagent (Invitrogen). To ensure equal amounts of plasmid in each transfection mixture, an empty expression vector was added as necessary. At 48 h post-transfection, cells were harvested, and protein extracts were subjected to a dual-luciferase assay using a commercially available kit (E1910; Promega) following the manufacturer’s instructions. Luminescence was measured with a GloMax 20/20 luminometer (E5331; Promega). The data shown are raw values of luciferase activity normalized to that of Renilla luciferase.

To analyze whether GLUT1 is potentially involved in the activation of β-catenin-dependent transcription, stimulated by virus productive infection, MDBK cells were cotransfected with the Super 8× TOPFlash plasmid and a plasmid encoding Renilla luciferase (Promega) using Lipofectamine 3000 Transfection Reagent. After transfection for 36 h, the cells were either mock-infected or infected with virus for 24 h in the presence of DMSO control or BAY-876 at the designated concentrations. Then the cells were harvested, and protein extracts were subjected to a dual-luciferase assay using a commercially available kit (E1910; Promega) according to the manufacturer’s instructions. Luminescence was measured with a GloMax 20/20 luminometer (E5331; Promega). The data shown are raw values of firefly luciferase activity normalized to that of Renilla luciferase.

### Infection of calves

Four-month-old female Holstein cows, which had not been vaccinated against BoAHV-1 and were seronegative for BoAHV-1 as determined using a commercial BoAHV-1 IgG indirect ELISA kit (BioStone Animal Health, Southlake, TX, USA, catalog number 10074-05), were utilized for these studies. The calves were inoculated in each nostril and eye with 1 mL of DMEM containing 1 × 10^7^ PFU of virus, without scarification, for a total of 4 × 10^7^ PFU per animal, as previously described (66). The calves were maintained under strict isolation conditions and were administered antibiotics before and after BoAHV-1 infection to prevent secondary bacterial infections. Nasal swabs and ocular swabs were collected every 2 days and frozen at −80°C for the detection of virus shedding by 50% endpoint titration method using MDBK cells, as described elsewhere (67).

The time point of acute infection was selected as 4 dpi. Latency was confirmed after approximately 60 days of infection, as evidenced by the absence of virus shedding in both ocular and nasal swabs, as previously described (66). Following euthanasia, the TG tissues were collected, sectioned into small pieces, and processed according to standard histopathological protocols, including formalin fixation and paraffin embedding.

### Detection of GLUT1 in TG neurons using immunofluorescence assay

TG tissues embedded in paraffin were cut into thin sections (10 μm), mounted on glass slides, and processed for IHC following standard protocols as described elsewhere (68, 69) with modification. In brief, slides containing TG sections were deparaffinized, hydrated, and peroxidase blocked through a series of washes that included (i) two xylene washes for 5 min each, (ii) two 100% ethanol washes for 2 min each, (iii) one 3% hydrogen peroxide wash (30% hydrogen peroxide was diluted 1:10 in 100% methanol) for 10 min, (iv) one 95% ethanol wash for 3 min, (v) one 70% ethanol wash for 1 min, and (vi) one double-distilled H2O wash for 1 min. After antigen retrieval using proteinase K, the slides were incubated overnight at 4°C in a humidified chamber with antibodies against GLUT1 (Abclonal, cat# A6982, diluted 1:50 in 1% BSA in PBST [PBS + 0.1% Tween-20]). After washing two times using PBS, secondary antibody incubation was then performed using Alexa Fluor 488-conjugated goat anti-rabbit IgG (H + L) (Invitrogen, cat# A-11,008, 1:1,500 dilution) and Alexa Fluor 633-conjugated goat anti-mouse IgG (H + L) (Invitrogen, cat# A-21052, 1:1,500 dilution) for 1 h in the dark at room temperature. After three additional washes, the nuclei were stained using DAPI (4′,6-diamidino-2-phenylindole). Slides were covered with coverslips by using antifade mounting medium (Electron Microscopy Sciences, cat# 50-247-04). Images were captured under light microscope (Nikon, Tokyo, Japan).

### Statistical analysis

All the data analyses were performed by Prism software 8.3 and IBM SPSS Statistics, version 25. All experimental data are presented as mean value ± standard deviation (SD). When comparing multiple groups, a one-way analysis of variance was applied. *P*-values below 0.05 were considered statistically significant.

## Data Availability

The data sets used and/or analyzed during the current study are available from the corresponding author on reasonable request.
